# Targeted Mutations in the Fusion Peptide Region of La Crosse Virus Attenuate Neuroinvasion and Confer Protection against Encephalitis

**DOI:** 10.3390/v14071464

**Published:** 2022-07-02

**Authors:** Bradley S. Hollidge, Mary-Virginia Salzano, John M. Ibrahim, Jonathan W. Fraser, Valentina Wagner, Nicole E. Leitner, Susan R. Weiss, Friedemann Weber, Francisco González-Scarano, Samantha S. Soldan

**Affiliations:** 1 Department of Neurology, University of Pennsylvania School of Medicine, Philadelphia, PA 19104, USA; bhollidge@regenexbio.com (B.S.H.); maryvirginia.salzano@almacgroup.com (M.-V.S.); ibrahimj@einstein.edu (J.M.I.); jonwfraser@gmail.com (J.W.F.); neleitner83@gmail.com (N.E.L.); weisssr@pennmedicine.upenn.edu (S.R.W.); scarano@mail.med.upenn.edu (F.G.-S.); 2 Neuroscience Graduate Group, University of Pennsylvania School of Medicine, Philadelphia, PA 19104, USA; 3 Abteilung Virologie, Institut für Medizinische Mikrobiologie und Hygiene, Universität Freiburg, 79008 Freiburg, Germany; valentina.wagner@uniklinik-freiburg.de (V.W.); friedemann.weber@vetmed.uni-giessen.de (F.W.); 4 Institute for Virology, FB10-Veterinary Medicine, Justus-Liebig University, 35392 Giessen, Germany; 5 The Wistar Institute, Philadelphia, PA 19104, USA

**Keywords:** La Crosse virus, fusion peptide, viral encephalitis, neuroinvasion, orthobunyavirus, *Bunyavirales*

## Abstract

La Crosse virus (LACV) is a major cause of pediatric encephalitis and aseptic meningitis in the Midwestern, Mid-Atlantic, and Southern United States, where it is an emerging pathogen. The LACV Gc glycoprotein plays a critical role in the neuropathogenesis of LACV encephalitis as the putative virus attachment protein. Previously, we identified and experimentally confirmed the location of the LACV fusion peptide within Gc and generated a panel of recombinant LACVs (rLACVs) containing mutations in the fusion peptide as well as the wild-type sequence. These rLACVs retained their ability to cause neuronal death in a primary embryonic rat neuronal culture system, despite decreased replication and fusion phenotypes. To test the role of the fusion peptide in vivo, we tested rLACVs in an age-dependent murine model of LACV encephalitis. When inoculated directly into the CNS of young adult mice (P28), the rLACV fusion peptide mutants were as neurovirulent as the rLACV engineered with a wild-type sequence, confirming the results obtained in tissue culture. In contrast, the fusion peptide mutant rLACVs were less neuroinvasive when suckling (P3) or weanling (P21) mice were inoculated peripherally, demonstrating that the LACV fusion peptide is a determinant of neuroinvasion, but not of neurovirulence. In a challenge experiment, we found that peripheral challenge of weanling (P21) mice with fusion peptide mutant rLACVs protected from a subsequent WT-LACV challenge, suggesting that mutations in the fusion peptide are an attractive target for generating live-attenuated virus vaccines. Importantly, the high degree of conservation of the fusion peptide amongst the *Bunyavirales* and, structurally, other arboviruses suggests that these findings are broadly applicable to viruses that use a class II fusion mechanism and cause neurologic disease.

## 1. Introduction

The *Bunyavirales* is the largest and arguably most diverse order of viruses, with more than 477 virus species currently described and further classified in fifteen families [1,2]. The *Bunyavirales* order was established in 2017 to accommodate the discovery of several viruses that could not be classified within the existing genera of the no-longer recognized *Bunyaviridae* family. Bunyaviruses are distributed worldwide and infect a broad range of invertebrate and vertebrate hosts. With the exception of hantaviruses and arenaviruses, all bunyaviruses are arthropod borne (arboviruses) and rely on transmission between hosts by invertebrate vectors. Some members of the *Orthobunyavirus, Phlebovirus,* and *Orthonairovirus* genera are neurotropic and infect the human central nervous system (CNS) and cause neurologic disease [3,4]. 

La Crosse virus (LACV; genus *Orthobunyavirus*, family *Peribunyaviridae*), a member of the California serogroup of orthobunyaviruses, is a leading cause of pediatric encephalitis and aseptic meningitis in the Midwestern United States and an emerging pathogen in the American South. Although an estimated 300,000 systemic LACV infections occur annually, only 50–150 cases of LACV encephalitis are reported per annum, predominantly in children younger than 16 years [5]. Nevertheless, it is increasingly appreciated that LACV can cause encephalitis in adults and can be associated with changes in mental status, intensive care unit admissions, and a need for post-discharge rehabilitation in those older than 16 [6]. Between 2009–2018, the Centers for Disease Control reported an average of 68 annual cases of pediatric La Crosse encephalitis, with most cases occurring in the states of Ohio, North Carolina, West Virginia, and Tennessee. It is widely believed that these cases—particularly the milder ones—are underreported [7]. Endemic transmission by its native vector, the Eastern treehole mosquito (*Ochlerotatus triseriatus*), is now exceeded by transmission by the invasive and aggressive day-biting Asian tiger mosquito (*Aedes albopictus*) and the Asian bush mosquito (*Aedes japonicus)*, which are at the vanguard of spread and emergence of arboviruses in the Southern United States [7,8]. 

Fever, vomiting, photophobia, and nausea are the most commonly reported symptoms of LACV infection [9]; less frequently, weakness, stiff neck, seizures, and coma are reported [9]. Approximately half of patients with LACV encephalitis develop seizures during the acute illness. Electroencephalographically, these are similar to those of herpes simplex encephalitis, including focal features or periodic lateralizing epileptiform discharges, typically involving the temporal lobe [8]. About 10% of survivors develop chronic epilepsy, and may continue to have neurological deficits that can include sixth-nerve palsies, hemiparesis, and neurobehavioral sequelae [8]. Interestingly, cognitive impairment, a high incidence of attention-deficit-hyperactivity disorder (60%), and poor academic performance are frequently observed following LACV encephalitis [10]. The case fatality rate of LACV encephalitis has been estimated to be between 1–3% [9,11]. Currently, there are no specific antivirals or vaccines for LACV. 

The three negative-sense, single-stranded RNA genome segments of LACV each have defined roles in virus pathogenesis. The S segment encodes a virulence factor, NSs, that efficiently inhibits the interferon system of infected mammalian hosts; its absence results in reduced virulence in mice [12,13,14]. In at least one instance, neurovirulence mapped to the L segment, which encodes the viral polymerase [15]. The neuroinvasive phenotype of LACV has been mapped to its M segment [16,17,18,19,20], which encodes the two viral glycoproteins, Gc and Gn. Gc, the larger of the two glycoproteins, serves as the viral attachment protein and is the exclusive target of anti-LACV neutralizing antibodies [21]. Recently, x-ray crystallography of three Orthobunyaviruses (Schmallenberg virus, Oropouche virus, and Bunyamwera virus) revealed the formation of spikes at the N-terminal extension of Gc [22]. Importantly, this spike appears to be the target of neutralizing antibodies and immunization with the Gc spike provides sterilizing immunity to mice, underscoring the importance of Gc in mediating Orthobunyavirus fusion and entry [22]. 

**Figure 1:**
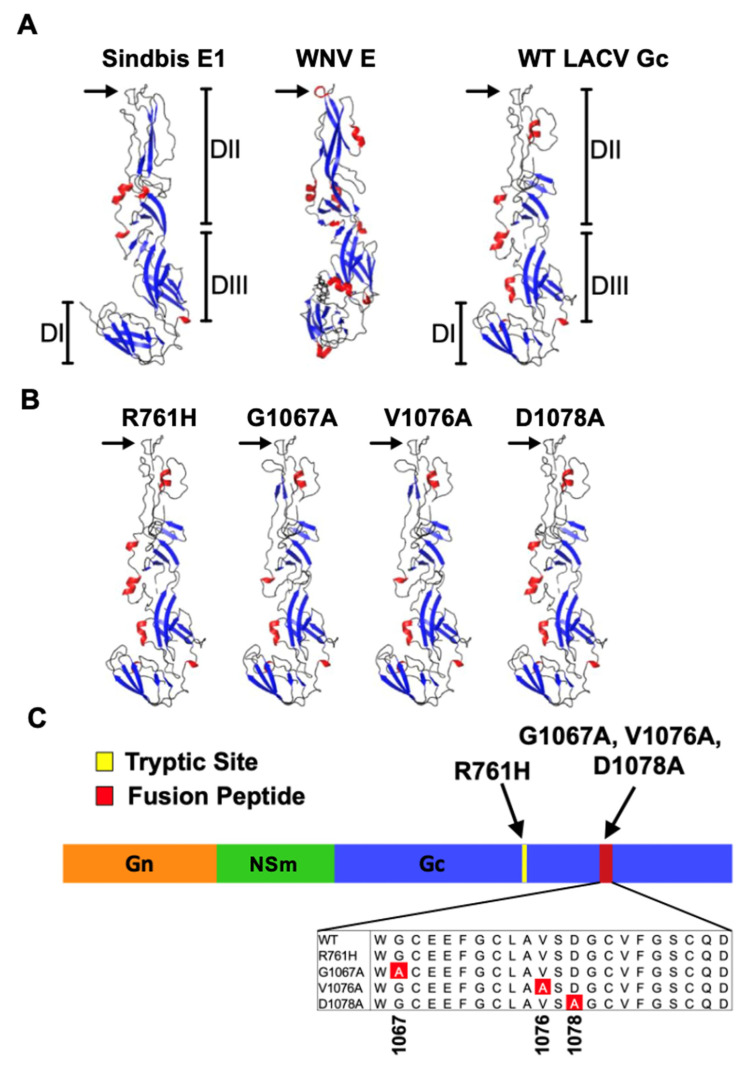
Structural models of WT-LACV Gc compared to other viral class II fusion glycoproteins, including Sindbis virus E1 and West Nile virus E based on SFV E1 as the canonical structure using Phyre2 and visualized with MacPyMOL. West Nile virus (WNV) E was included as reference. Structural models demonstrate secondary structure as ß-strands (blue), α-helices (red), and (hydrophobic) loops (gray). The fusion loop of each glycoprotein is labeled with a black arrow. Domain I (DI) is a ß-barrel with two long extensions connecting adjacent ß-strands. Domain II (DII) contains the highly conserved fusion peptide loop at its tip (black arrows) connecting the c and d ß-strands of DII. (**A**) Flexible hinge region is located between DI and DII. Domain III (DIII) is a stem region connecting the fusion protein to the transmembrane anchor. In the post fusion hairpin structure, DIII and the fusion loop are brought into close proximity. (**B**) Structural models predict that the mutations in fusion peptide of LACV Gc do not alter the structure of Gc or the fusion loop. (**C**) Schematic representing the locations of the mutations within Gc and within the highly conserved fusion peptide.

We demonstrated that the proximal two-thirds of Gc, amino acids 860-1442, are critical in LACV fusion and entry [21,23,24]. Using computational analysis, we identified structural similarities within this region of LACV Gc (amino acids 970-1350) to the E1 fusion protein of two alphaviruses, Sindbis virus and Semliki Forrest virus (SFV) [23,25]. Collectively, these studies determined that LACV Gc functions as a class II membrane fusion protein, akin to the alphavirus E1 glycoprotein and the flavivirus E glycoprotein (Figure 1A). Furthermore, a 22-amino acid hydrophobic segment (1066-1087) within the aforementioned proximal two-thirds was predicted to correlate structurally with a hydrophobic domain of Semliki Forrest virus (SFV) and Sindbis virus [23,25]. Importantly, this region is highly conserved among the *Bunyavirales* and the placement of its cysteine residues resembles those of the fusion domains of SFV E1 and sarcoma-leukosis virus E, where the cysteine resides form an internal loop [26]. Using site-directed mutagenesis of conserved residues within LACV Gc 1066-1087, we created mutant M-segment constructs to demonstrate that LACV Gc 1066-1087 functions as the LACV fusion peptide in a luciferase-based, cell-to-cell fusion assay, and in a pseudotype transduction assay [27]. From this panel of M-segment constructs, we generated recombinant LACVs (rLACVs) with mutations in the fusion peptide domain using a three-cDNA plasmid, reverse-genetics system [28]. Five rLACVs were rescued: three with mutations within the fusion peptide domain (G1067A, V1076A and D1078A) (Figure 1B,C), one with a mutation in a site highly susceptible to digestion with trypsin outside the fusion peptide domain (R761H, site not shown), and a control recombinant with the wild-type (WT) LACV sequence. The fusion peptide domain mutant rLACVs (G1067A, V1076A, and D1078A) were growth-impaired in muscle cells, insect cells, and primary rat neuronal cultures and had diminished fusion phenotypes. However, the fusion peptide mutant rLACVs remained as neurotoxic as WT virus in a cell-based, microtubule associated-protein-2 (MAP-2, a neuronal marker) ELISA, using neuronal cultures prepared from embryonic day 17 Sprague Dawley rat pups, despite impaired growth and fusion of these viruses [28]. These data suggested that the LACV fusion peptide is associated with properties of neuroinvasion (growth to high titer in muscle cells and robust fusion), but not of neurovirulence [28].

Here, we used a murine model of LACV encephalitis that mirrors many aspects of the human disease, including its age-dependent susceptibility, to study the LACV fusion peptide as a viral determinant of CNS invasion and/or virulence [17,29]. The age-dependent neuroinvasiveness of LACV is associated with its ability to generate a high viremia, which has been found to correlate with virus replication in striated muscle [17]. Importantly, even after intracranial inoculation, there are age-related differences in viral antigen in the brain; suckling mouse brains demonstrate disseminated viral antigen, while viral antigen is more restricted to specific neuroanatomical regions in adult mice, particularly sparing the white matter [17]. In this model, neuroinvasiveness is defined by neurological disease following peripheral inoculation in suckling (P3) or weanling mice (P21), whereas neurovirulence is defined by infection directly into the brain of young adult mice (P28) [29]. Weanling (P21) and young adult (P28) mice remain susceptible to subcutaneous inoculation with LACV at higher titers, but become increasingly less susceptible with age [17]. Recent studies using older adult mice (P42-56) have further demonstrated that brain capillary endothelial cells from adult mice have reduced viral loads, infectivity, and susceptibility to LACV infection, which influences the age-dependent susceptibility to LACV-mediated BBB leakage and CNS disease [29]. 

In the present study, congruent with our in vitro findings, we found that the fusion peptide domain mutant rLACVs had delayed neuroinvasion in suckling (P3) and weanling (P21, not shown) mice inoculated peripherally, whereas neurovirulence was not affected when rLACVs were inoculated directly into the CNS of young adult mice (P28). These data suggest that a time-dependent increase in viral load, likely driven by virus replication in muscle, is critical for efficient virus entry in the CNS. In the weanling (P21) mice challenged intraperitoneally, these mutant viruses also conferred protection from subsequent wild-type LACV (WT-LACV) challenge. Collectively, this study demonstrates that the fusion peptide of LACV is a determinant of neuroinvasion, but not of neurovirulence. These studies underscore the potential use of recombinant viruses with targeted mutations in the fusion peptide as the basis of live-attenuated, neuroprotective virus vaccines.

## 2. Materials and Methods

### 2.1. Cells and Viruses

Vero cells were grown at 37 °C, 5% CO_2_ in Dulbecco’s modified Eagle’s medium (DMEM) supplemented with 10% fetal bovine serum (FBS; Atlanta Biologicals, Atlanta, GA, USA), 1% (*v*/*v*) penicillin/streptomycin (Invitrogen, Carlsbad, CA, USA), 1% (*v*/*v*) L-glutamine (Invitrogen, Carlsbad, CA, USA), and 1% sodium pyruvate. BSR-T7/5 cells were also cultivated in DMEM supplemented with 10% fetal calf serum and 1 mg/mL geneticin [30]. The origin and preparation of wild-type LACV have been described [17]. 

### 2.2. Generation of Fusion Peptide Mutant Viruses

Recombinant LACVs were generated from Gc mutant constructs as described [27] using a reverse genetics system [28,30]. Briefly, subconfluent layers of BSR-T7/5 cells were transfected using Fugene transfection reagent (Roche, Pleasanton, CA, USA) with 0.5 µg of each plasmid encoding a genome segment of LACV (pT7Ribo-LACV-cL, pT7Ribo-LACV-cSNoEco, and pT7Ribo-LACV-cM (or variants thereof)) as cDNA. Five days later, supernatants of transfected wells were harvested and 200 µL were used to inoculate Vero cells for 1 h at 37 °C followed by replacement of fresh medium. The appearance of a cytopathic effect was monitored in these Vero cells for the next five days. 

### 2.3. Inoculation of Mice and Harvest of Tissues

Outbred albino Swiss mice (CD-1; Charles River Laboratories, Wilmington, MA, USA) were used for all experiments. Suckling (72 h old) mice were inoculated in the footpad while under isofluorane anesthesia with rLACVs diluted in PBS/0.75% bovine serum albumin (BSA; 20 µL volume) or control diluent. Young adult mice (P28) were inoculated intracranially with rLACV diluted in PBS/BSA or control diluent while anesthetized. For rechallenge studies in weanling mice (P21), the initial rLACV challenge and subsequent rLACV boosts or WT-LACV rechallenges were administered intraperitoneally in a final volume of 200 µL of PBS/BSA. Mice were weighed daily and monitored twice daily (disease in this model can progress rapidly) for signs of disease including seizures, limb paralysis, unbalanced gait, and ruffled fur. Mice exhibiting clinical disease or moribund mice were promptly euthanized. Following euthanasia, tissues (axillary lymph nodes, heart, lung, liver, kidney, quadriceps muscle, spleen, spinal cord, and the left hemisphere of the brain (right hemisphere fixed for immunohistochemistry)) were collected individually in Dulbecco’s Modified Eagle Medium (DMEM; Gibco) supplemented with 2% fetal bovine serum (Atlanta Biologicals, Atlanta, GA, USA), 1% (*v*/*v*) penicillin/streptomycin (Invitrogen, Carlsbad, CA, USA), 1% (*v*/*v*) L-glutamine, and 1% (*v*/*v*) sodium pyruvate and frozen at −20 °C. Prior to tissue titrations, tissues were thawed at 37 °C, homogenized, and centrifuged for 10 min at 1200 rpm to remove cell debris. Additionally, blood was collected by cardiac puncture immediately following euthanasia or by retro-orbital bleed. The serum was separated for titration and detection of anti-LACV antibodies. These experiments were approved by the University of Pennsylvania Institutional Animal Care and Use Committee.

### 2.4. Plaque Assays

Vero cells were used for plaque assays to determine viral titers in mouse tissues. Cells were plated at 1.5 × 10^5^ cells/well in a 12-well plate and incubated overnight at 37 °C, 5% CO_2_. Ten-fold serial dilutions of tissue homogenates or serum were used to infect VERO monolayers in duplicate for 1 h. The homogenates were removed and a 1:1 mixture of 2% carboxymethyl-cellulose (Sigma-Aldrich, Saint Louis, MO, USA) and 2X DMEM supplemented with 4% fetal calf serum, 2% (*v*/*v*) penicillin/streptomycin (Invitrogen, Carlsbad, CA, USA), 2% (*v*/*v*) L-glutamine, and 2% (*v*/*v*) sodium pyruvate. Carboxymethyl-cellulose overlays were removed after 72 h and plaques were visualized by staining with 0.1% crystal violet. 

### 2.5. Neutralization Assay 

Neutralizing antibody titers were quantified in sera collected from weanling (P21) mice by a plaque reduction neutralization titer (PRNT_50_). Serial dilutions starting at 1:10 were prepared in DMEM (Invitrogen, Carlsbad, CA, USA) supplemented with 4% fetal calf serum. LACV was diluted to a final titer of 400 PFU/mL and was added to equal volumes of the serum dilutions and mixed well. LACV was also added to a 1:100 dilution of the LACV neutralizing monoclonal antibody 807.31 as a positive control. The serum/virus mixture was incubated at 37 °C for 60 min, added to confluent monolayers of Vero cells, and incubated for 1 h to allow virus attachment. Cells were overlaid with 2% carboxymethyl-cellulose and incubated for 5 days at 37 °C. After incubation, the overlay was removed, and the monolayers were washed twice with PBS and stained with crystal violet to allow for the enumeration of virus plaques. A 50% plaque-reduction neutralization titer was calculated using standard methodology (Reed-Muench method).

### 2.6. Histopathology and Immunohistochemical Staining

For immunohistochemical analysis, the right hemispheres of harvested brains were fixed in phosphate-buffered formalin. Brains were embedded in paraffin, sectioned at 5 µm and mounted onto slides by the Wistar Institute Histotechnology Facility. Two sections per brain were stained with hematoxylin and eosin (H&E). Slides were incubated overnight at 55 °C, then deparaffinized with xylene followed by rehydration in a series of ethanol solutions (100%, 95%, 90%, and 70%) and water. 

For fluorescent immunohistochemical staining, rehydrated sections were permeabilized in 3% H_2_O_2_ (1:10 dilution of 30% H_2_O_2_ solution) in methanol for 30 min. Antigen was retrieved by citric acid-based antigen unmasking solution (Vector Laboratories, Burlingame, CA, USA) during 10 min of boiling. Slides were blocked in 10% normal goat serum in PBS overnight at 4 °C and then incubated ovenight at 4 °C in rabbit or chicken antibodies diluted in 10% normal goat serum in PBS. Anti-rabbit or anti-chicken antibodies were diluted in PBS and incubated for 1 h at 37 °C. Primary mouse antibodies were used with the Vector Laboratories mouse-on-mouse immunodetection kit. Primary antibodies used were rabbit anti-LACV (1:50), chicken anti-MAP2 (1:5000; Abcam, Waltham, MA, USA), chicken anti-GFAP (1:1000; Abcam, Waltham, MA, USA), mouse anti-GFAP (1:100; Cell Signaling, Danvers, MA, USA), rabbit anti-cleaved-caspase-3 (1:300; Cell Signaling, Danvers, MA, USA), and rabbit anti-cleaved PARP (1:200; Cell Signaling, Danvers, MA, USA). All secondary antibodies and Hoecht’s stain were used at a 1:100 dilution. Detection of apoptosis in brain sections was performed using NeuroTACS II in situ apoptosis detection kit (Trevigen, Gaithersburg, MD, USA) following manufacturer’s instructions. 

### 2.7. Statistical Analysis

Kaplan–Meier survival curves were analyzed by the long-rank (Mantel–Cox) test. The statistical significance of all other data was evaluated by unpaired two-tailed Student’s *t*-tests or Mann–Whitney tests. A *p*-value of <0.05 was considered significant. All data are expressed as the mean ± standard error of the mean. Statistical analysis was performed using GraphPad Prism software. 

## 3. Results

### 3.1. Neurovirulence and Neuroinvasiveness of rLACVs in the Murine Model of LACV Encephalitis

**Figure 2:**
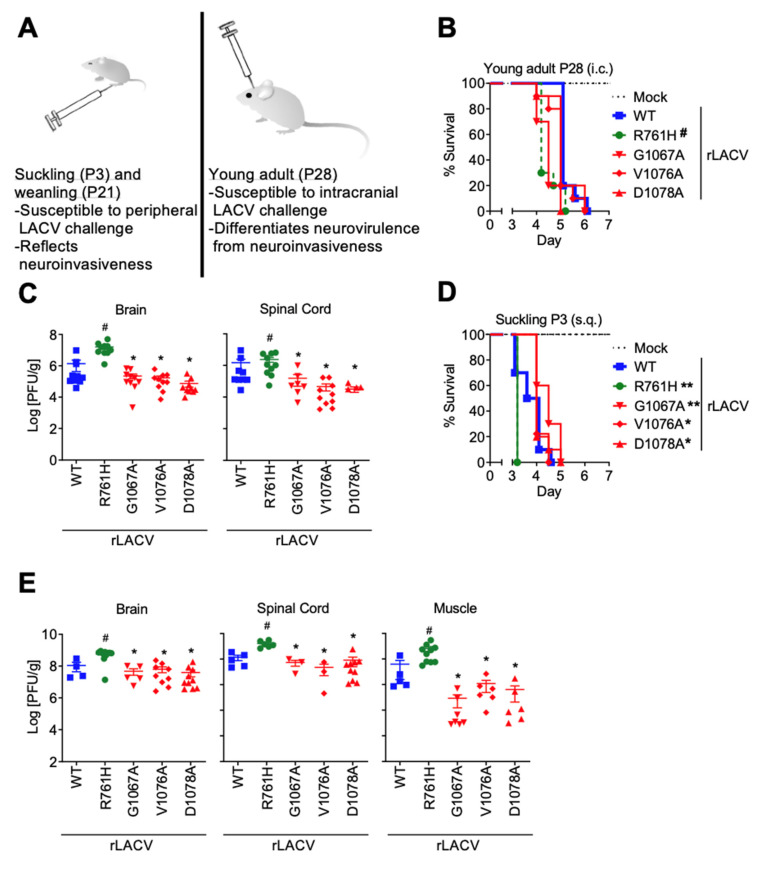
Fusion peptide mutant rLACVs have lower viral loads in key tissues and are less neuroinvasive, but remain neurovirulent in vivo. (**A**) The murine model of LACV encephalitis differentiates determinants of neuroinvasion from neurovirulence. (**B**) Four-week-old mice were inoculated intracranially with 10 PFU of rLACV-WT, the tryptic site mutant (rLACV-R761H), or the fusion peptide domain mutants rLACVs (G1067A, V1076A, and D1078A). There were no significant differences in the survival rates of young adult (P28) mice inoculated with rLACV-WT compared to those inoculated with the fusion peptide mutant rLACVs. Interestingly, young adult (P28) mice inoculated with the tryptic site mutant virus died significantly earlier than mice inoculated with rLACV-WT (Log-rank (Mantel–Cox) test; # *p* < 0.001; *n* = 10 per group). (**C**) Fusion peptide mutant viruses grew to lower titers in the brains and spinal cords of young adult (P28) mice compared to rLACV-WT (* *p* < 0.05). The tryptic site mutant rLACV grew to higher titers in the brains and spinal cords (# *p* < 0.05 increase). (**D**) Suckling (P3) mice were inoculated subcutaneously with 5 PFU of the rLACVs (*n* = 10 per group, except *n* = 9 for V1076A). Suckling (P3) mice challenged with the fusion peptide mutant viruses (rLACV-G1067A, rLACV-V1076A, and rLACV-D1078A) survived longer than those inoculated with rLACV-WT. As with the young adult (P28) mice, suckling (P3) mice challenged with the tryptic site mutant rLACV-R761H died significantly earlier than mice inoculated with rLACV-WT (Log-rank (Mantel–Cox) test; * *p* < 0.05; ** *p* < 0.01). (**E**) Fusion peptide mutant rLACVs grew to lower titers in the brains, spinal cords, and muscle of suckling mice challenged subcutaneously (* *p* < 0.05; # *p* < 0.001).

In our murine model of LACV encephalitis (Figure 2A), young adult (P28) mice are susceptible to intracranial (i.c.) challenge with WT-LACV and used to determine factors associated with neurovirulence. We inoculated young adult (P28) mice intracranially with 10 PFU of rLACV-WT, rLACV-761H, or one of the fusion peptide domain mutants (rLACV-G1067A, rLACV-V1076A, and rLACV-D1078A). Kaplan–Meier curves compared the survival of mice inoculated with rLACV-WT with the survival of mice inoculated with either the fusion peptide mutant viruses (rLACV-G1067A, rLACV-V1076A, and rLACV-D1078A) or the tryptic site mutant rLACV (R761H). The survival of young adult (P28) mice inoculated with fusion peptide mutant viruses was not statistically different from those inoculated with rLACV-WT (Figure 2B; Log-Rank (Mantel–Cox) test). Similar survival curves were generated when mice were inoculated with 100 PFU (data not shown). All mice that succumbed to disease during the study displayed signs of encephalitis, including seizures, limb paralysis, unbalanced gait, and ruffled fur. Therefore, the fusion peptide does not determine neurovirulence once LACV reaches the CNS. Interestingly, seizures were observed more frequently in suckling (P3) mice inoculated subcutaneously (s.c.) compared to young adult (P28) mice inoculated i.c. (Table 1). Inoculation with rLACV-R761H, which has a mutation at position 761, and therefore, outside the fusion peptide of LACV Gc, resulted in a significant enhancement of neurovirulence compared to rLACV-WT (Figure 2B; *p* < 0.001; Log-Rank (Mantel–Cox) test). This position is uniquely accessible to trypsin in the intact Gc molecule [28,31,32]. Additionally, the viral loads in brain and spinal cord of the mice challenged with this tryptic site mutant rLACV (R761H) were significantly higher in comparison with the brains and spinal cords of mice challenged with rLACV-WT (Figure 2C; *p* < 0.05). In contrast, the viral titers from the brains and spinal cords of mice challenged with the fusion peptide domain mutant rLACVs were significantly lower than those of mice challenged with rLACV-WT (Figure 2C; *p* < 0.05). Additionally, the tryptic site mutant rLACV (R761H) was the only rLACV consistently recovered outside the CNS of these mice with virus detected in the liver and spleen (Supplementary Table S1).

In contrast to young adult (P28) mice, suckling mice (72 h old) are extremely susceptible to peripheral challenge with low doses (<1 PFU) of WT-LACV (Figure 2A). Peripheral challenge differentiates viral factors associated with neuroinvasion. Suckling mice (P3) were inoculated via the footpad with either 5 PFU of rLACV-WT, rLACV-761H, or one of the fusion peptide domain mutants. Kaplan–Meier curves comparing their survival are shown in Figure 2D. Suckling mice challenged with the fusion peptide domain mutant viruses survived significantly longer than mice challenged with rLACV WT (Figure 2D; *p* < 0.05 for rLACV-V1076A, and rLACV-D1078A; *p* < 0.01 for rLACV-G1067A). Similar survival curves were generated when mice were inoculated with 10 PFU (data not shown). Note that although the endpoints are similar, there is a delay in the onset of mortality, consistent with the neurovirulence assessments (once the virus is in the brain, the mice do not survive). As with the young adult (P28) mice, the end-point viral titers were significantly lower in the brains and spinal cords of suckling mice challenged with the fusion peptide domain mutant viruses in comparison with mice challenged with rLACV-WT (Figure 2E; *p* < 0.05), which may be linked to lower replication of fusion peptide mutant viruses. In addition, the viral titers of the three fusion mutants were significantly lower in muscle, which is the primary site of replication in the periphery and required for the virus to reach a high viremia, than after inoculation with rLACV-WT (Figure 2E; *p* < 0.05). In contrast, the tryptic site mutant virus (rLACV-R761H) reached significantly higher viral titers in the CNS as well as muscle (Figure 2E; *p* < 0.05). Therefore, these mutations in the fusion peptide domain of LACV impair virus replication in the CNS and in peripheral tissues. Together, these studies demonstrate the LACV fusion peptide is associated with neuroinvasion without a concomitant attenuation of neurovirulence. 

### 3.2. Neuropathology of rLACVs

**Figure 3:**
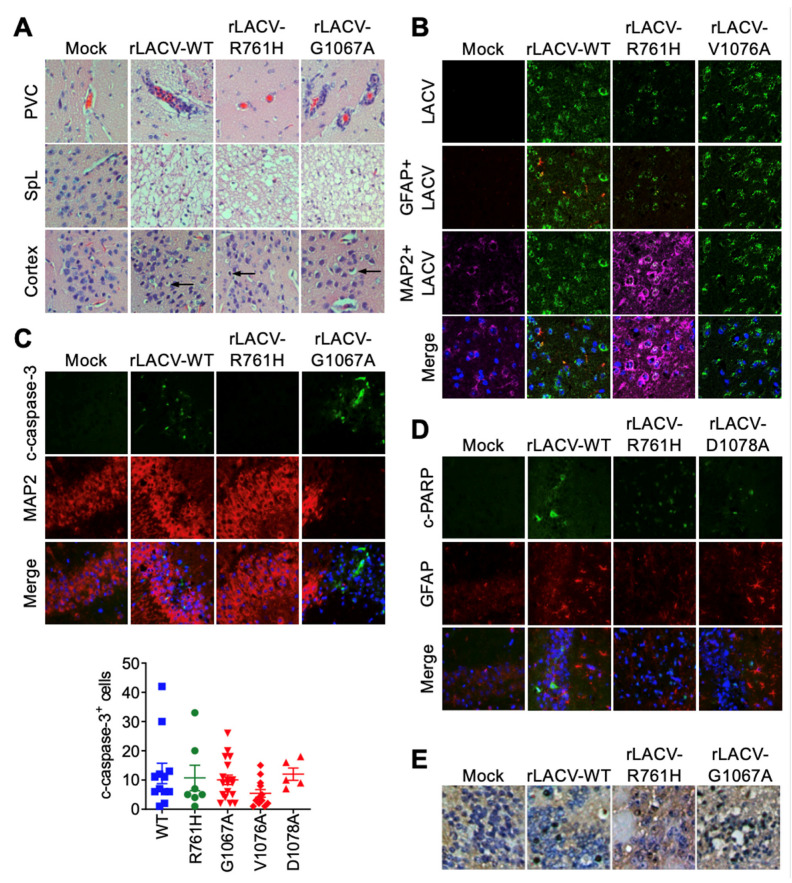
Histopathological changes and viral antigen in the brains of rLACV-infected, four-week-old mice challenged intracranially. (**A**) Hematoxylin and eosin staining of brains of moribund, four-week-old mice challenged with rLACVs showing perivascular cuffs (PVC), spongiform lesions (SpL), and degenerating neurons (arrows) in the cortex. (**B**) Immunofluorescence of LACV antigen (Green) in the dorsal thalamus (ventral to the hippocampus) of moribund mice (GFAP = Red; MAP2 = Purple; Nuclei = Hoecht’s, blue) demonstrating neuronal localization. (**C**) Cleaved-caspase-3 (Green) and MAP2 (Red) immunofluorescence in CA3 of the hippocampus of moribund mice. (**D**) Cleaved-PARP (Green) and GFAP (Red) immunofluorescence in CA1 of the hippocampus of moribund mice. (**E**) TUNEL-positive degenerating neurons (dark blue) and LACV antigen with hematoxylin counterstain.

Histologic examination was limited to the brains of moribund mice to compare lesions produced in the CNS due to rLACV infections. In young adult (P28) mice challenged intracranially with rLACVs, perivascular cuffing was present in the brains of mice inoculated with rLACV-WT and the fusion peptide domain mutants (rLACV-G1067A, rLACV-V1076A, and rLACV-D1078A; rLACV-G1067A shown as a representative example) (Figure 3A). Interestingly, there was minimal perivascular cuffing in mice challenged with the tryptic site mutant (rLACV-R761H) (Figure 3A). Additionally, all rLACVs resulted in spongiform lesions in the brain though the size and locations of these lesions varied (Figure 3A). The cerebral cortex of rLACV infected young adult (P28) mice showed only sparse neuronal degradation (Figure 3A,E). Colocalization of LACV antigen with MAP2 (a neuronal marker) demonstrated that the rLACVs primarily infected neurons, but not astrocytes (GFAP; an astrocyte marker), of the hippocampus, thalamus, hypothalamus, and midbrain with more dispersed LACV antigen in the cortex of mice challenged with rLACVs (Figure 3B). The finding that neurons are the primary targets of LACV in the brain is consistent with previous reports [33]. Cleaved-caspase-3 (Figure 3C) and cleaved-PARP (Figure 3D) were present in the brains of moribund mice with dispersed staining in similar regions to where LACV antigen was present, as were TUNEL-positive cells (Figure 3E), indicating widespread neuronal death. Notably, cleaved-caspase 3 is not always associated with death in neurons. However, the presence of cleaved-caspase-3, cleaved-PARP, and TUNEL staining is suggestive of neuronal apoptosis [34].

**Figure 4:**
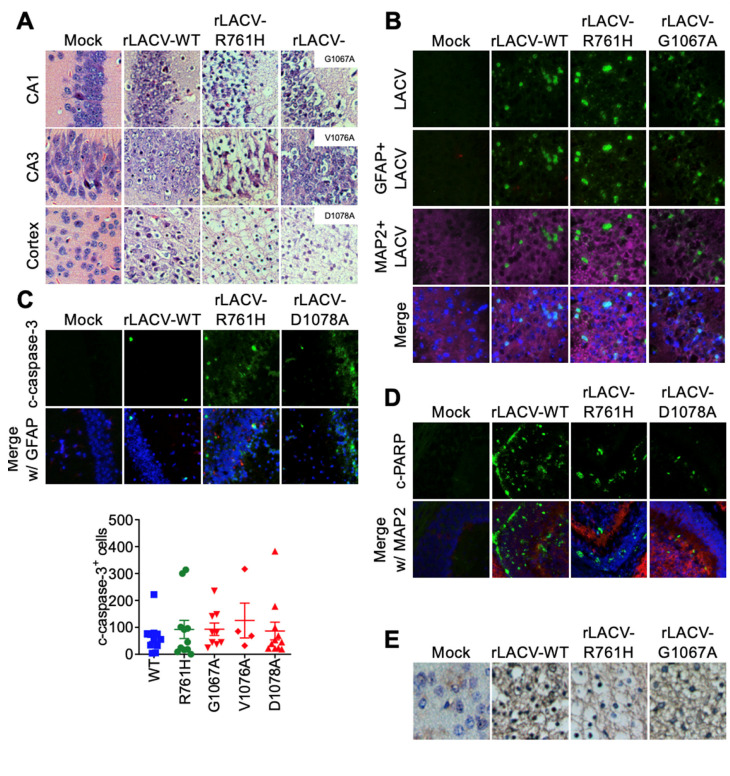
Histopathological changes and viral antigen in the brains of rLACV infected suckling mice challenged subcutaneously. (**A**) Hematoxylin and eosin staining showed widespread neuronal degeneration in the suckling mouse brains after rLACV infection. (**B**) Immunofluorescence of LACV antigen (Green) in the CA3 region of the hippocampus of moribund mice (GFAP = Red; MAP2 = Purple; Nuclei = Hoecht’s, blue). (**C**) Cleaved-caspase-3 (Green) and GFAP (Red) immunofluorescence in the hippocampus of moribund mice. (**D**) Cleaved-PARP (Green) and MAP2 (Red) immunofluorescence in the cerebellum of moribund mice. (**E**) TUNEL-positive degenerating neurons (dark blue) and LACV antigen with hematoxylin counterstain.

In contrast to young adult (P28) mice, suckling (P3) mice showed widespread neurodegeneration, particularly in the cortex (Figure 4A). However, no perivascular cuffing was present in the suckling mice challenged with the rLACVs. LACV antigen was disseminated throughout the brain, including the cerebellum and hippocampus with neurons being the main cell type expressing LACV antigen (Figure 4B). There were apoptotic cells throughout the brain, including the cerebellum (Figure 4C–E); however, there were fewer apoptotic cells in the hippocampus in comparison with the cerebellum (Figure 4C), which may be due to the extensive neuronal loss already sustained by the hippocampus earlier in the CNS infection (Figure 4A,B). TUNEL-positive cells were present throughout the brain of sucking mice where LACV antigen was present (Figure 4E).

### 3.3. Attenuated Fusion Peptide Mutant rLACVs Are Protective against WT-LACV Rechallenge

**Figure 5:**
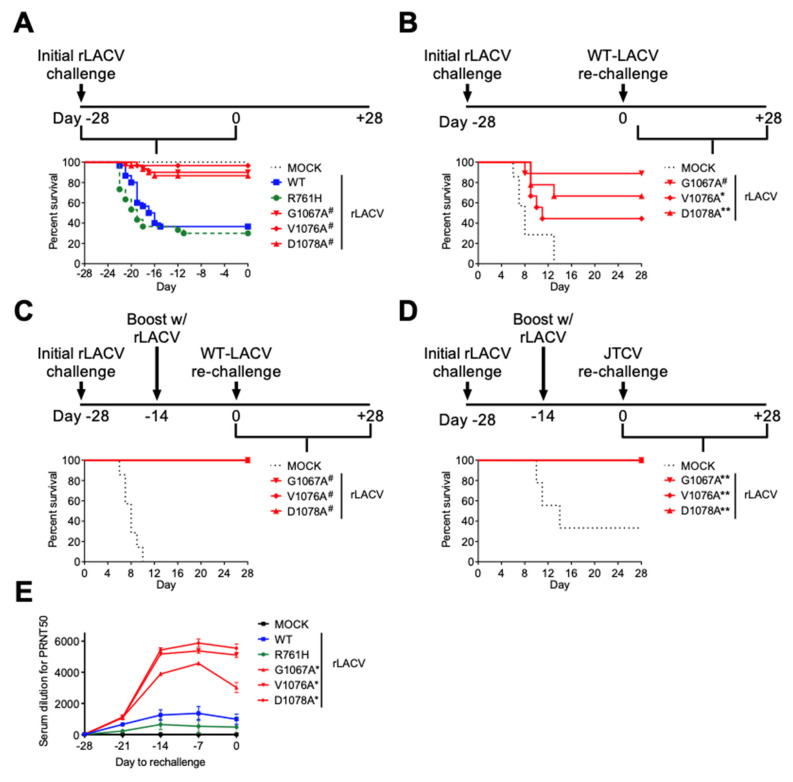
Fusion peptide domain mutant rLACVs are less neuroinvasive and protect against subsequent WT-LACV rechallenge. (**A**) Three-week-old mice were inoculated intraperitoneally with 1000 PFU of rLACV-WT, the tryptic site mutant (rLACV-R761H), or the fusion peptide domain mutant rLACVs (G1067A, V1076A, and D1078A). The mice challenged with the fusion peptide domain mutants had a significantly increased survival rate compared to rLACV-WT (Log-rank (Mantel–Cox) test; *n* = 30 per group). (**B**) Three-week-old mice infected with 1000 PFU were challenged 28 days after rLACV inoculation with 100*LD_50_ of WT-LACV intraperitoneally. Mice infected with rLACV-G1067A were almost fully protected and mice infected with rLACV-V1076A or rLACV-D1078A were partially protected from lethal WT-LACV challenge. (**C**,**D**) Three-week-old mice immunized with 1000 PFU of rLACVs were boosted with 1000 PFU of the same rLACV 14 days following initial rLACV challenge. Fourteen days following rLACV boosts, the mice were challenged with 100×LD_50_ of (**C**) WT-LACV or (**D**) Jamestown Canyon virus intraperitoneally. Mice immunized and boosted with the fusion peptide domain mutant rLACVs were fully protected from lethal WT-LACV or Jamestown Canyon virus rechallenge. (* *p* < 0.05; ** *p* < 0.01; # *p* < 0.001; Log-rank (Mantel–Cox) Test). (**E**) Neutralizing antibodies measured by PRNT_50_.

To assess whether the recombinant fusion peptide domain mutants elicit a protective immune response against subsequent challenge with a lethal dose of WT-LACV, we inoculated weanling (P21) mice, which are less susceptible to peripheral challenge of LACV, via the intraperitoneal route, with 1000 PFU of the rLACVs. Challenge with fusion peptide domain mutant rLACVs resulted in increased survival compared to rLACV-WT inoculated mice (Figure 5A; Survival: 30/30 Mock; 11/30 rLACV-WT; 9/30 rLACV-R761H; 27/30 rLACV-G1067A; 29/30 rLACV-V1076A; 26/30 rLACV-D1078A; *p* < 0.001). These data support our conclusion that the fusion peptide domain mutant viruses are less neuroinvasive than rLACV-WT. The mice were rechallenged with 100×LD_50_ (8 × 10^5^ PFU) of WT-LACV intraperitoneally (i.p.) 28 days following initial challenge with the rLACVs. An immunizing dose of 100 PFU of the fusion peptide mutant rLACVs provided only partial protection from a lethal WT-LACV challenge (not shown), while an immunizing dose of 1000 PFU of the fusion peptide mutant rLACVs resulted in a greater protection (Figure 5B; Survival: 0/7 Mock; 8/9 rLACV-G1067A; 4/9 rLACV-V1076A; 6/9 rLACV-D1078A; * *p* < 0.05; ** *p* < 0.01; # *p* < 0.001). However, full protection from a lethal WT-LACV challenge was achieved through an initial i.p. immunization of 1000 PFU of the fusion peptide mutant rLACVs followed by an i.p. boost of another 1000 PFU 14 days after the initial immunization. The WT-LACV challenge then took place 14 days after the boost (Figure 5C; Survival: 0/7 Mock; 6/6 rLACV-G1067A; 6/6 rLACV-V1076A; 5/5 rLACV-D1078A; # *p* < 0.001). Neutralizing antibodies generated to LACV have been shown to cross-react with other California serogroup bunyaviruses, especially Snowshoe Hare and Tahyna viruses, but also to a lesser extent with Jamestown Canyon (JCV) and Inkoo viruses [35]. Therefore, it was of interest to determine whether inoculation with fusion peptide mutant rLACVs conferred protection against another California serogroup virus. We found that an immunizing dose of 1000 PFU with a similar boost provided mice full protection from lethal challenge with JCV (Figure 5D; Survival: 3/9 Mock; 8/8 rLACV-G1067A; 10/10 rLACV-V1076A; 8/8 rLACV-D1078A; ** *p* < 0.01). PRNT analysis demonstrated that animals challenged with fusion peptide domain mutant rLACVs had significantly higher neutralizing antibody titers compared to mice initially challenged with WT rLACV or with the tryptic peptide mutant (Figure 5E).

## 4. Discussion

Reverse-genetics systems to generate rLACVs (and other negative-sense RNA viruses) are an important advance in studying virus pathogenesis and, potentially, the development of antiviral treatment modalities and vaccines. Additionally, targeting highly conserved regions in the virus genome could provide viral targets for attenuation across viral genera or families that use similar mechanisms. Previously, we used a three-cDNA plasmid, reverse-genetics system to rescue rLACVs with mutations in the highly conserved fusion peptide to study this region in the context of a virus infection. Four mutant rLACVs were rescued, three with single mutations in the fusion peptide domain (rLACV-G1067A, rLACV-V1076A, and rLACV-D1078A) and one with a mutation in a tryptic site (rLACV-R761H) (Figure 1) [27]. An rLACV with the wild-type genome was rescued as a control (rLACV-WT). These rLACVs represent a range of predicted fusion phenotypes [27]: two with normal fusion phenotypes (rLACV-WT and rLACV-R761H), one with moderately impaired fusion phenotype (rLACV-G1067A), and two with severely impaired fusion phenotypes (rLACV-V1076A and rLACV-D1078A) [27]. Interestingly, the rLACV with moderately impaired fusion (rLACV-G1067A) generated less robust neutralizing antibody responses than the two with severely impaired fusion phenotypes (Figure 5E).

**Figure 6:**
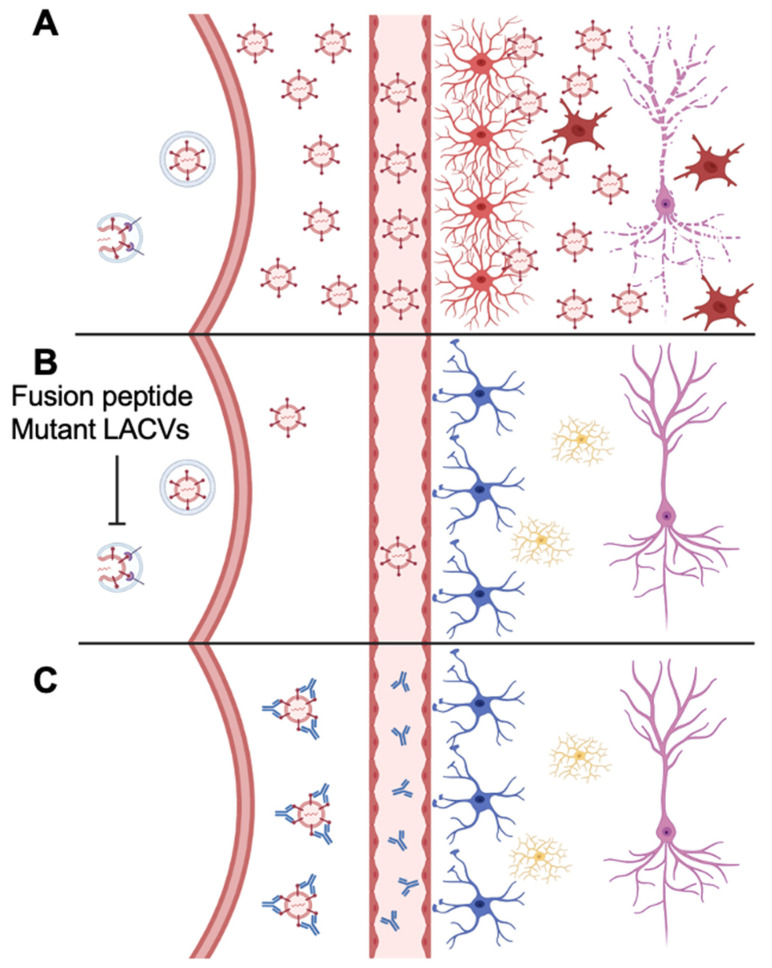
Model of neuroprotection for rLACV rechallenge. (**A**) WT LACV or WT rLACV robustly replicates in muscle resulting in viremia. Virus enters the CNS causing neurologic disease characterized by neuronal damage, reactive astrocytes, and activated microglia. (**B**) Fusion peptide mutants rLACVs replicate to a lower level in muscle resulting in less virus in the CNS and decreased neurologic disease. (**C**) After infection with fusion peptide mutant rLACVs, neutralizing antibodies are produced, which decrease replication of WT LACV in the muscle after rechallenge preventing virus from entering the CNS and preventing neurologic disease.

We had previously determined that mutants with reduced fusion function, such as LACV mAb escape variants V22 and V22F (generated with 807-22, a mAb against LACV Gc), replicated to lower levels than wild-type virus in the peripheral tissues (e.g., striated muscle; Figure 6) that are critical for the generation of a high viremia. Whereas V22 and V22F had an impaired ability to mediate cell-to-cell fusion, their mutations were not localized to the fusion peptide region. Although V22 and V22F were less neuroinvasive than WT-LACV, owing to the limited viremia, they were as virulent as WT-LACV when introduced directly into the CNS of mice [36]. The present experiments tested the hypothesis that more precise mutations targeting the putative fusion domain would also have similar phenotypes. In fact, the in vitro studies with the fusion peptide domain mutants demonstrated that these viruses had impaired growth in differentiated muscle cells and primary rat neuronal cultures as well as impaired fusion phenotypes, but remained neurotoxic [28]. Collectively, these studies emphasize that the ability of a virus to replicate to high titer in muscle is a major determinant of efficient neuroinvasion and spread.

We then challenged young adult mice (P28) intracranially and suckling (P3) mice peripherally with the rLACVs, differentiating the properties of neuroinvasivenss and neurovirulence, as previously described [36]. When the rLACVs were injected directly into the CNS of young adult (P28) mice, the survival curves of mice challenged with fusion peptide domain mutant rLACVs were not significantly different from those challenged with rLACV-WT (Figure 2B), suggesting these viruses retained their neurovirulence in vivo. Nevertheless, the viral titers of the fusion peptide mutant rLACVs in the brains and spinal cords of these mice were significantly lower than those of mice challenged with rLACV-WT (Figure 2C). These results are consistent with our in vitro studies [28]. Therefore, both the in vitro and in vivo results suggest the fusion peptide of LACV is not a determinant of neurovirulence and these viruses do not need to reach wild-type levels to cause fatal CNS disease in mice.

Peripheral challenge of suckling mice (72 h old) was then used to assess the neuroinvasiveness of the rLACVs. While all of the suckling (P3) mice died regardless of which rLACV they were challenged with, the mice inoculated with the fusion peptide domain mutant rLACVs had a significantly longer survival compared to rLACV-WT challenged mice, suggesting that the fusion peptide is a determinant the neuroinvasive potential of LACV. Replication in striated muscle is one of the primary factors of neuroinvasion by enabling virus amplification and the generation of a high viremia [20]. Indeed, viral titers in the striated muscle of mice inoculated with the fusion peptide domain mutants (rLACV-G1067A, rLACV-V1076A, and rLACV-D1078A) were significantly reduced compared to titers in the muscle of mice infected with rLACV-WT (Figure 2E); this reduced viral titer in muscle corresponded with increased survival in suckling (P3) mice inoculated subcutaneously (Figure 2E). Importantly, reduced replication of fusion peptide mutants was also observed in the brains and spinal cords of young adult mice (P28) inoculated intracranially, demonstrating reduced proliferation of the fusion peptide mutants in the CNS may also contribute to the delayed disease course observed in the suckling (P3) model. Furthermore, the role of the fusion peptide as a determinant of neuroinvasion is supported by our rechallenge studies (Figure 5). Only 8 of 90 weanling mice (3/30 G1067A; 1/30 V1076A; 4/30 D1078A) that were initially challenged by the fusion peptide domain mutants intraperitoneally succumbed to LACV infection (Figure 5A). As with the young adult (P28) mice challenged intracranially (Figure 2C), the suckling (P3) mice challenged with the fusion peptide domain mutant viruses had significantly lower viral titers in the CNS compared to mice challenged with rLACV-WT (Figure 2E). 

Interestingly, infection with the tryptic site mutant (rLACV-R761H), which was generated as a control because it has a mutation in Gc outside the LACV fusion peptide domain, resulted in significantly shorter survival times of both young adult (P28) mice inoculated intracranially (Figure 2B) and suckling (P3) mice challenged via the footpad (Figure 2D). Additionally, the viral titers in tissues of the CNS as well as periphery (in the suckling (P3) mice) were significantly higher for rLACV-R761H than those of rLACV-WT (Figure 2C,E). Therefore, the tryptic site mutant has increased neuroinvasiveness as well as neurovirulence in this murine model. The Gc 761 is only one of many potential proteolytic sites in Gc [31], yet is uniquely accessible on the virion prior to acidification with its accessibility being affected by a low-pH induced conformational change [32]. In theory, this mutation could weaken the interaction of Gn and Gc. The induction of Gc trimerization may result from the exposure of the fusion loop or a strengthened interaction with the Gc monomers prior to trimerization. Alternatively, the R761H mutation may increase the flexibility of the hinge region between domains I and II leading to a more flexible hairpin reaction, making the postfusion hairpin a more energetically favorable conformation with increased efficiency. Another possibility is that the tryptic site mutation may alter the conformation outside of the fusion reaction by altering post-translational modifications and/or processing, possibly even trypsin cleavage, to make Gc a more stable glycoprotein. Further studies are required to determine the mechanism of rLACV-R761H’s altered virulence pattern. 

The neuropathology of the tryptic site mutant (rLACV-R761H) was also different from rLACV-WT and the fusion peptide domain mutants. Young adult (P28) mice challenged with any of the rLACVs showed spongiform lesions as well as single cell or focal necrosis, and apoptotic bodies. However, brains of mice challenged with the tryptic site mutant rLACV-R761H did not show any perivascular cuffing (Figure 3A), which may result from the increased viral titers in the brains of rLACV-R761H inoculated mice and suggests that these mice succumb to disease before the onset of encephalitis/inflammation. These data may indicate that the neurovirulence of rLACV-R761H is more virus-mediated rather than a result of inflammation. However, in the suckling mice, infection with all of the rLACVs resulted in overwhelming neuronal necrosis with little concomitant inflammation, suggesting that suckling mice are also primarily succumbing directly to virus-mediated neuronal destruction (Figure 4A). Additionally, introducing these mutations into LACV did not alter the cellular tropism of these viruses in the CNS, with neurons being the primary cell type infected. 

The susceptibility and neuropathology observed in of suckling mice could be due to an underdeveloped innate immune system, resulting in the inability to control peripheral LACV infection resulting in a high viremia. For example, suckling mice were only able to produce small amounts of interferon in only their livers when infected with Coxsackie B1 virus, while young adult (P28) mice produced interferons in all tissues that became infected [37]. Additionally, macrophage maturation may be another underdeveloped immune component in suckling mice responsible for their susceptibility to LACV infection, as demonstrated for herpes simplex virus type 2 [38]. Therefore, the suckling immune system may be unable to prevent the peripheral replication and spread of LACV. 

For our rechallenge studies, weanling mice (P21) were used because they are less susceptible to peripheral challenge with LACV compared to suckling (P3) mice. Our studies showed that the fusion peptide domain mutant rLACVs provided partial protection from WT-LACV rechallenge (Figure 5). However, providing a boost with the same rLACVs provided full protection from WT-LACV or JCV rechallenge. 

Virus fusion peptides may provide a novel target for attenuation of viruses for vaccines or antiviral strategies. Monoclonal against the fusion peptide of influenza A virus have been shown to neutralize multiple subtypes of the virus by inhibiting the pH-dependent fusion of viral and cellular membranes [39]; mAb 1C9 (HA2 domain of influenza A H5) protected 100% of mice from a lethal dose of highly pathogenic avian influenza H5N1 viruses form two different clades [40]. The abrogation of virus fusion by mutagenesis of the fusion peptide would likely not result in enough virus replication/production of virus antigen to elicit a strong enough antibody response to protect against rechallenge. Additionally, for a safe and effective vaccine, there would have to be concomitant attenuation of other virulence factors, especially since these rLACV fusion peptide mutants are still neurovirulent (Figure 2B). This study demonstrates that single mutations in the fusion peptide can result in rLACVs that are attenuated enough to diminish neuroinvasion, yet replicate well enough for weanling mice to generate protective immune responses, especially when provided with a boost prior to WT-LACV rechallenge (Figure 5). Notably, these fusion peptide mutant rLACVs are also cross protective against JTCV challenge (Figure 5D). Therefore, our previous in vitro studies may serve as a rationale to generate variants of LACV, and other similar viruses, for attenuated neuroinvasiveness and/or neurovirulence. Importantly, because the fusion peptide region is conserved among the *Bunyavirales*, the findings from these studies may be extrapolated to other emerging bunyaviruses, including Crimean-Congo hemorrhagic fever virus and Rift Valley fever virus with the potential to lead to the development of therapeutics and vaccines. 

## Figures and Tables

**Table 1:** Frequency of seizures observed in suckling and young adult mice challenged s.c. (suckling) or i.c. (young adult) with rLACVs.

rLACV	Suckling (P3)	Young Adult (P28)
WT	40%	11%
R761H	40%	15%
G1067A	70%	26%
V1076A	33%	15%
D1078A	70%	20%

## Data Availability

Data available upon request.

## Supplementary Materials

The following supporting information can be downloaded at: www.mdpi.com/article/10.3390/v14071464/s1. Table S1: frequency of rLACV recovery outside the CNS.
