# Supplementary material for: Targeted Mutations in the Fusion Peptide Region of La Crosse Virus Attenuate Neuroinvasion and Confer Protection against Encephalitis
Source: Viruses. 2022 Jul 2;14(7):1464. doi: 10.3390/v14071464 (PMC9317099; doi:10.3390/v14071464)
Supplement: Supplementary file 1 [file viruses-14-01464-s001.zip › viruses-1686095-supplementary.pdf]

Supplemental Table S1

rLACV	Heart	Lung	Liver	Kidney	Quadriceps	Spleen	Lymph Node
WT	0/10	0/10	0/10	0/10	0/10	0/10	0/10
R761H	0/10	0/10	3/10	0/10	0/10	2/10	0/10
G1067A	0/10	0/10	0/10	0/10	0/10	1/10	0/10
V1076A	0/10	0/10	0/10	0/10	0/10	0/10	0/10
D1078A	0/10	0/10	0/10	0/10	0/10	0/10	0/10
